# The dual antimicrobial and immunomodulatory roles of host defense peptides and their applications in animal production

**DOI:** 10.1186/s40104-022-00796-y

**Published:** 2022-12-06

**Authors:** Di Wu, Linglong Fu, Weizhang Wen, Na Dong

**Affiliations:** State Key Laboratory of Animal Nutrition, College of Animal Science and Technology, China Agricultural University, Beijing, 100193 China

**Keywords:** Antimicrobials, Host defense peptides, Immunomodulation, Poultry, Ruminants, Swine

## Abstract

Host defense peptides (HDPs) are small molecules with broad-spectrum antimicrobial activities against infectious bacteria, viruses, and fungi. Increasing evidence suggests that HDPs can also indirectly protect hosts by modulating their immune responses. Due to these dual roles, HDPs have been considered one of the most promising antibiotic substitutes to improve growth performance, intestinal health, and immunity in farm animals. This review describes the antimicrobial and immunomodulatory roles of host defense peptides and their recent applications in animal production.

## Introduction

Antibiotics have been used in animal production for more than 50 years [1]. However, the extensive use of antibiotics in animal production has resulted in widespread antimicrobial resistance by pathogens. Antibiotic residues in animal-derived foods transfer antibiotic resistant bacteria to humans, meaning that antimicrobial resistance has become a great threat to human health [2]. Novel and effective antibiotic substitutes are therefore urgently needed.

Host defense peptides (HDPs), also known as antimicrobial peptides (AMPs), are essential components of the innate immune system and exist in virtually all forms of life, ranging from single-celled bacteria to multi-cellular organisms [3]. In recent years, HDPs have been studied as antibiotic substitutes due to their potent antibacterial activities against Gram-positive, Gram-negative, and multidrug-resistant bacteria [4]. HDPs principally interact with the bacterial membrane and destroy membrane integrity, but they additionally act on intracellular targets to interfere with key cellular processes [5, 6]. HDPs modulate both innate and adaptive immune responses by regulating inflammation, recruiting leukocytes, activating immune cells, and modulating adaptive immunity, all of which help to protect the host against bacterial infection [7–9]. A key advantage of HDPs over conventional antibiotics is the ability of HDPs to modulate host immunity in response to microbial infection.

HDPs have received increasing attention for potential use in animal production due to their antimicrobial and immunomodulatory roles. Many HDPs can positively influence growth performance, nutrient digestibility, intestinal health, and immune function in animals. This review focuses on the antimicrobial and immunomodulatory roles of HDPs and their applications in livestock development.

## HDPs

HDPs are evolutionarily conserved molecules, generally comprising 10–50 amino acids, that are found among nearly all forms of life that have defense functions [10]. More than 3000 HDPs have been identified to date and are hosted in the Antimicrobial Peptide Database (https://aps.unmc.edu/AP/main.php). Among the more than 3000 known HDPs, 2431 originate from animals, 361 from plants, 371 from bacteria, 5 from archaea, 8 from protists, 22 from fungi, and some from synthetic peptides.

HDPs are divided into three categories based on their origin: animal HDPs, plant HDPs, and bacterial HDPs. Based on structural differences and biological characteristics, animal HDPs can be further classified into two subfamilies: cathelicidins and defensins (including α-defensin and β-defensin). Among the known animal HDPs, human cathelicidin LL-37 is the most well-studied [11]. Plant HDPs are widely distributed; they are found in stems, flowers, leaves, roots, and seeds, and include thionins, defensins, hevein‑like peptides, knottin‑type peptides, lipid transfer proteins, and snakins. These plant HDPs defend against bacteria, fungi, and insects[12]. Bacterial HDPs, also called bacteriocins, play important roles in regulating competitive interactions in natural microbial systems. Some are narrow-spectrum, efficient antibacterial compounds, and these characteristics contribute to their potential to limit or prevent colonization by diarrheal pathogens [13, 14]. In addition to natural HDPs, an increasing number of synthetic HDPs have been reported in recent years. Compared with the natural HDPs, synthetic peptides have optimized sequences that confer low resistance to digestive enzymes and minimal cell cytotoxicity, circumventing the drawbacks of natural HDPs [15]. HDPs are highly diverse in sequence and structure but can be classified into four major structural groups: α-helical (e.g., melittin and cecropins), β-sheet (e.g., α- and β-defensins), β-hairpin (e.g., lactoferricin and tachyplesins), and extended HDPs (e.g., indolicidin and histatins) [5, 10]. Regardless of the natural source or structure, all HDPs can kill pathogens and modulate host immune responses.

## Antimicrobial roles of HDPs

The unique ability of HDPs to kill pathogenic bacteria provides a platform for researchers to develop promising alternatives to antibiotics.

### Broad-spectrum antimicrobial activities

Numerous researchers have shown that HDPs possess broad-spectrum antimicrobial activity against bacteria (Gram-negative, Gram-positive, and drug-resistant), fungi, and viruses [16, 17]. For example, human defensing (hBD)-3 was shown to have antimicrobial activity against several pathogenic bacteria, such as *Staphylococcus aureus*, *Streptococcus pyogenes*, *Pseudomonas aeruginosa*, *Escherichia coli* (*E. coli*), multidrug-resistant *S. aureus*, and vancomycin-resistant *Enterococcus faecium*, in addition to the fungal pathogen *Candida albicans* [18]. Wang et al. isolated 12 novel HDPs from frogs, and most showed potent antimicrobial activities against Gram-positive bacteria (*E. coli*, *Enterococcus faecalis*, and *Enterobacter cloacae*), Gram-negative bacteria (*S*. *aureus*, *Klebsiella pneumonia*, and *Bacillus dysenteriae*), and a fungus (*C. albicans*) [19]. Bovine myeloid HDPs, a group of α-helical cathelicidins, exhibited powerful inhibitory effects against antibiotic-resistant species such as methicillin-resistant *S. aureus* (MRSA), vancomycin-resistant *E. faecalis* (VREF), and multidrug-resistant strains of *P. aeruginosa* and *Acinetobacter baumannii* [20]. Subtilosin displays potent antiviral activity against herpes simplex virus type 1 and 2 [21, 22].

### Antibacterial mechanisms

The antibacterial mechanisms of HDPs are diverse and dependent on the properties of individual HDPs and the microbial pathogens upon which they are acting. Based on their interacting sites, the antibacterial mechanisms of HDPs can be divided into membrane targeting and non-membrane targeting mechanisms.

#### Membrane targeting mechanism

Cell membranes are widely considered to be the main site of antibacterial action for HDPs [5]. Bacterial membranes are negatively charged due to the presence of anionic phospholipids, namely lipopolysaccharides (LPS) in Gram-negative bacteria and teichoic acids (TA) in Gram-positive bacteria [7]. Most HDPs are rich in positively-charged residues such as lysine, arginine, and histidine [16, 23]. Therefore, the electrostatic interactions between bacterial membranes and HDPs lead to the initial targeting of cell membranes, although HDPs also later attract more peptides [24]. The efficiency of this process is strongly related to the cationic charge and concentration of HDPs that are bound to the membrane interface. Generally, increased binding activity results from more highly charged HDPs and higher HDP concentrations (within a physiological range) [25]. At higher concentrations of HDPs, interactions between the peptides and membranes tend to depend on hydrophobicity, which controls the extent to which peptides can penetrate the membrane layer [26, 27]. When the number of HDPs attached to the membrane reaches a critical concentration, self-association, multimerization, and conformational transformation occur. This change eventually causes membrane penetration and disruption; the specifics of this process have been described by various hypothetical models, such as the carpet, aggregate, toroidal pore, and barrel-stave models [28]. Membrane penetration eventually leads to leakage of intracellular ions and metabolites, ultimately causing cell death. In contrast to conventional antibiotics, which require days to be effective, HDPs cause bacterial death within minutes of exposure [29].

#### Non-membrane targeting mechanism

In addition to inducing perturbation of bacterial membranes, HDPs can kill bacteria by inhibiting metabolic and translational processes, such as protein synthesis, nucleic acid synthesis, and enzyme activity [30]. For example, proline-rich HDPs can bind to the ribosomal exit tunnel and subsequently prevent protein synthesis [31]. The histone-derived HDPs buforin II and desHDAP1 interact with the phosphate groups of DNA via hydrogen bonding, altering DNA conformation and function and thus inhibiting bacterial growth [32]. NP-6, an HDP derived from pepper seeds, targets *E. coli* by inhibiting β-galactosidase activity in a dose-dependent manner[33].

## Immunomodulatory roles of HDPs

In animals, HDPs are often located at sites where environmental pathogen exposure is most likely to occur, such as the skin, ears, eyes, epithelial surfaces, lungs, and gut, but also in the bone marrow, testes, ovary and oviduct [34, 35]. Invasion by pathogens can induce expression of host HDP genes at early stages of infection, which facilitates elimination of bacteria by the host [36]. HDPs expression is likely a prophylactic response to infection. Immunomodulatory activities of HDPs are more extensive than the antimicrobial activities and seem to depend on the degree and phase of bacterial infection, the physiological status of host cells, and the HDP concentration[37, 38]. Understanding the mechanisms of HDPs immunomodulation will be helpful in determining practical applications of HDPs in animal production.

### Modulation of inflammation

Inflammation is a biological response of the innate immune system to defend against invading pathogens. However, overwhelming and uncontrolled inflammation can cause severe injury to the host [39]. HDPs exhibit both pro- and anti-inflammatory roles. For example, they can up-regulate inflammatory factors to activate the immune system, which helps to eliminate invading pathogens early in an infection. This is considered a pro-inflammatory response. Conversely, HDPs can suppress over-reactive inflammatory responses induced by bacteria or bacterial products. This is considered an anti-inflammatory response. Therefore, HDPs can modulate inflammation to maintain immune homeostasis.

Bactenecin-5 and epinecidin can induce transcription of interleukin (IL)-1β in the presence and absence of live *Mycobacterium marinum* in macrophage-like THP-1 cells. Bactenecin-5 was also found to significantly up-regulate tumor necrosis factor-α (TNF-α), but the pro-inflammatory activity of bactenecin-5 required co-stimulation with *M. marinum* [40]. Oral administration of sublancin, an HDP derived from *Bacillus subtilis*, restored expression of IL-2, IL-4, and IL-6 in immunosuppressed mice and accelerated recovery of phagocytic activity by macrophages [41]. These results indicate that HDPs can activate immune responses by inducing the release of pro-inflammatory cytokines.

LPS is a major component of Gram-negative bacterial outer membranes, and can be recognized by host toll like receptor-4 (TLR4), and activates production of pro-inflammatory cytokines by immune cells via TLR4 signaling [42]. The anti-inflammatory functions of HDPs are mainly due to LPS-neutralizing activity, which suppresses downstream TLR4 signaling pathways (such as mitogen-activated protein kinase [MAPK] and nuclear factor-κB [NF-κB] signaling) [43, 44]. For example, a frog-derived peptide, cathelicidin-MH, exerts LPS-neutralizing activity. This protects against LPS-induced sepsis in mice and significantly decreases production of the pro-inflammatory cytokines IL-1β, IL-6, and TNF-α by suppressing MAPK signaling [34]. Lactoferricin downregulated the secretion of the pro-inflammatory cytokines TNF-α, IL-6 in LPS-treated macrophages by targeting the MAPK and NF-κB pathways [45]. In addition to direct binding of HDPs to LPS, some HDPs (such as pigeon-derived cathelicidin Cl-CATH2 and 3, snake-derived cathelicidin Hc-CATH) bind to the opening region of the LPS-binding pocket on myeloid differentiation factor-2 (MD-2) of the TLR4-MD-2 complex in macrophages challenged by LPS. This direct binding inhibits activation of the TLR4 pathway induced by LPS, which in turn decreases expression of pro-inflammatory cytokines at the transcriptional level [46, 47].

### Recruitment of leukocytes

HDPs exhibit direct chemotactic activity towards leukocytes (such as neutrophils, macrophages, mast cells, and T cells). The underlying mechanisms involve several cellular receptors, including chemokine receptors, formyl peptide receptors (FPRs), and G protein-coupled receptors (GPCRs) [42]. hBD-3 had been reported to utilize C–C chemokine receptor type 2 (CCR2) to induce monocyte/macrophage chemotaxis [48]. The HDPs scolopendrasin and LL-37 recruit neutrophils, monocytes, and T cells to sites of bacterial infection by interacting with FPR1 and formyl peptide receptor-like 1 (FPRL1), respectively [49, 50]. G-protein pathways are reportedly involved in mast cell chemotaxis induced by the synthetic cationic HDP IDR-1018, which is associated with increased intracellular Ca^2+^ mobilization [51].

HDPs also indirectly facilitate recruitment of leukocytes by inducing production of chemokines and chemokine receptors. Cathelicidins can induce chemokine receptor CCR2, CXCR2, IFNγ-R, MRC1, and LFA1 production in monocytes. Additionally, stimulated monocytes produce various chemokines like CCL2, CCL5, CCL7, CXCL10, and CXCL8 (IL-8) [52]. HDP-IBP5, derived from insulin-like growth factor-binding protein 5, induces production of cytokines and chemokines such as granulocyte–macrophage colony-stimulating factor (GM-CSF), TNF-α, IL-8, MCP-1, MCP-3, macrophage inflammatory protein (MIP)-1α, and MIP-1β, which regulate migration of mast cells in a dose-dependent manner [53].

In vivo experiments have also demonstrated chemotaxis of HDPs. Inoculating mouse peritoneum with the scorpion-derived HDP ToAP2A increased levels of peritoneal macrophages and induced a greater chemotactic migration of neutrophils (and possibly eosinophils) [54]. Injection of PopuCATH (cathelicidin from tree frog) significantly elicited chemokines (CXCL1, CXCL2, and CXCL3)/cytokine (IL-1β and IL-6) production in macrophages through activating p38/ERK MAPKs and NF-κB p65 pathway, and rapidly drove neutrophil, monocyte/macrophage influx in mouse abdominal cavity [55].

### Activation of immune cells

Activation of immune cells by HDPs directly increased the bactericidal activities of these peptides and promoted early clearance of infections. Two synthetic peptides, Pin2[G] and FA1, stimulate phagocytosis of *Salmonella typhimurium* by macrophages [56]. LL-37 and hBD-2 enhance the expression and induce translocation of NOD1, NOD2, and RIG-I innate immunity receptors, and also directly activate the pro-inflammatory and migratory responses of peritoneal mast cells in vitro [57]. PopuCATH significantly enhanced neutrophil phagocytosis via promoting the release of neutrophil extracellular traps [55].

Immune cell activation by HDPs may be mediated by some immune cell receptors. Human host defense peptides, such as LL-37, and IBP5, can activate mast cells via the MAS-related G protein-coupled receptor X2 (MRGPRX2), which is highly expressed on mast cells and responds to various exogenous and endogenous stimuli. Activation of mast cells leads to degranulation, release of eicosanoids, and multicellular signaling cascades [53, 58]. Murine β-defensin-2 (mDF2beta) can directly activate immature dendritic cells by acting as an endogenous ligand for TLR4, resulting in up-regulation of costimulatory molecules and maturation of dendritic cells [59].

### Regulation of adaptive immunity

B and T cells are important participants in adaptive immunity and can influence the generation and polarization of lymphocyte responses. Stimulating resting porcine lymphocytes with nisin produced by *Lactococcus lactis* increased the percentage of CD4^+^CD8^+^ T cells. This effect may result from modulation of the stimulating potential of antigen-presenting cells [60]. Pathogenic stimulation significantly increased expression of cathelicidin genes in trout IgM^+^ and IgT^+^ B cells both in vitro and in vivo. Interestingly, these peptides increased the intracellular bactericidal, phagocytic, and reactive oxygen species (ROS) activities of trout IgM^+^ and IgT^+^ B cells [61]. Antigen-presenting cells (e.g., monocytes) can take up and process antigens and present them to T cells in concert with major histocompatibility complex (MHC) II molecules on the cell surface. Chicken cathelicidin-2 increases the antigen presentation capacity of chicken monocytes by up-regulating expression of the antigen presentation markers MHC-II and mannose receptor C-type 1 (MRC1) [62]. A similar effect on mouse macrophages was described in response to treatment with a chicken HDPs fowlicidin-1 [63]. Thus, HDPs improve antigen presentation capacity, which prepares antigen-presenting cells to function in an enhanced adaptive immune response against infection.

## Applications of HDPs in animal production

HDPs are evolutionarily conserved components of the innate host defense system that are present in essentially all forms of life. Farm animals produce a variety of endogenous HDPs in gut, mainly cathelicidins and defensins [64–67]. Supplementing exogenous HDPs could mimic the physiological release of endogenous HDPs and thus improve the host immune response against bacterial infections [68–70]. In recent years, HDPs have been used as alternatives to antibiotics to improve animal growth performance, immunity, and intestinal health; they have also been used as novel therapeutic agents to reduce the frequency and severity of subclinical infections (Tables 1 and 2).

**Table 1:** Effects of host defense peptides (HDPs) on swine

HDPs	Dose	Method of administration	Swine	Effects on growth performance (compared to control), %			Other effects (compared to control)	References
				ADG	ADFI	G/F		
Microcin J25	500 mg/kg 1000 mg/kg 2000 mg/kg	Feeding	Weaned pigs	+ 1.1 + 3.7 + 9.2	– 0.4 + 0.5 – 0.3	+ 1.7 + 3.4 + 6.9	Decreased the concentrations of the cytokines IL-6, IL-1β, and TNF-α and increased IL-10 level on serum, decreased *D*-lactate, diamine oxidase, and endotoxin concentrations and fecal *E. coli* numbers, improved fecal *Lactobacillus* and *Bifidobacterium* numbers	[71]
Cecropin AD	400 mg/kg	Feeding	Weaned pigs challenged with *E. coli*	+ 14.7	+ 4.6	+ 10.9	Decreased diarrhea incidence by 47.6%, improved villus height to crypt depth ratio in the jejunum and ileum, decreased total viable counts of *E. coli* while increased the *Lactobacilli* counts in cecum	[72]
Composite HDPs	500 mg/kg	Feeding	Weaned pigs	—	—	—	Increased the serum levels of immunoglobulin IgG, IgM, IgA and classical swine fever antibody CSF-Ab, CH50	[73]
HDP-WK3	2000 mg/kg BW	Injecting	Weaned pigs challenged with *E. coli*	+ 55.9	+ 28.9	+ 8.7	Decreased diarrheal index by 24.4%, increased villus height in the ileum, reduced numbers of *Enterobacterium *spp. in cecal and attenuated intestinal oxidative damage	[74]
Cathelicidin-BF	0.6 mg/kg BW	Injecting	Diarrheal weaned piglets	+ 70.3	+ 51.7	+ 11.1	Decreased diarrheal index by 40.1%, increased the expression levels of zonula occluden-1, Occludin, and Claudin-1 in the jejunum and colon, decreased IL-6, IL-8, IL-22, IL-10 production in the jejunum and ileum	[75]
Composite HDPs	4000 mg/kg	Feeding	Weaned pigs challenged with DON	+ 11.4	– 3.4	+ 17.5	Improved peripheral lymphocyte proliferation rate, serum antioxidant capacity, intestinal morphology, intestinal epithelial cell proliferation and protein synthesis, alleviate organ damage induced by DON	[76]
Epinecidin-1	2500 mg/kg BW	Injecting	Pigs challenged with MRSA	—	—	—	Decreased MRSA counts in the blood, liver, kidney, heart, and lungs, attenuated the levels of proinflammatory cytokine IL-6, IL-1β, and TNF-α in serum and MRSA-induced gene expressions	[77]

**Table 2:** Effects of host defense peptides (HDPs) on poultry

HDPs	Dose	Method of administration	Poultry	Effects on growth performance (compared to control), %			Other effects (compared to control)	References
				ADG	ADFI	G/F		
HDP-cLF36	20 mg/kg	Feeding	Chickens challenged with *E. coli*	+ 9.2	– 1.7	+ 11.6	Increased the number of *Lactobacillus *spp. and decreased harmful bacteria of ileum, upregulated gene expression of immune cells and tight junction proteins	[78]
Microcin J25	0.5 mg/kg 1.0 mg/kg	Feeding	Chickens challenged with *E. coli* and *Salmonella*	+ 2.5 + 2.6	+ 2.2 + 2.9	+ 3.4 + 3.9	Decreased population of total anaerobic bacteria and *E. coli*, increased the number of *Bifidobacterium*, increased the villus height and villus height/crypt depth in the duodenum and jejunum, decreased levels of TNF-α, IL-1β, and IL-6 in the serum	[79]
HDP-cLFchimera	20 mg/kg	Feeding	Chickens challenged with *C. perfringens*	+ 6.4	– 15.3	+ 26.0	Enhanced villus height, width, and surface area on jejunum, regulated the expression of cytokines, junctional proteins, and mucin transcripts in the jejunum, increased the population of *Lactobacillus *spp. and *Bifdobacterium *spp*.* and also decreased the colonization of *E. coli* and *Clostridium *spp*.* in ileum	[80]
Sublancin	2.88 mg/L 5.76 mg/L 11.52 mg/L	Drinking	Chickens challenged with *C. perfringens*	+ 10.4 + 12.3 + 10.4	+ 3.5 + 3.0 – 0.7	+ 7.3 + 9.1 + 10.9	Reduced the severity of intestinal lesion, reduced *C. perfringens* counts in cecum, improved villus height and villus height to crypt depth ratio in the duodenum, decreased IL-1β, IL-6, and TNF-α levels in ileum	[81]
HDP-SGAMP	0.2 mg/d	Gavaging	Chickens under heat stress	—	—	—	Reduced the histological and ultrastructural lesions of gut, increased height of villus and thickness of gut mucosa, increased the number of intestine intraepithelial lymphocytes and goblet cells, secreting IgA in the small intestine	[82]
Plectasin	150 mg/kg	Feeding	Chickens under heat stress	—	—	—	Increased jejunal and ileal goblet cell counts, IFN-γ levels and serum IgY titer	[83]
HDP-A3	60 mg/kg 90 mg/kg	Feeding	Chickens	+ 1.3 + 4.2	+ 0.2 + 1.8	+ 1.1 + 2.3	Increased the retention of dry matter, gross energy and crude protein, decreased excreta coliforms, total anaerobic bacteria and *Clostridium *spp., increased villus height of the duodenum, jejunum and ileum	[84]
Plectasin	100 mg/kg 200 mg/kg	Feeding	Chickens	+ 16.1 + 17.4	– 4.7 – 5.6	+ 21.8 + 24.5	Enhance NDV and H9N2 AIV antibody levels of serum, improved the intestine structure, inhibit *E. coli* and proinflammatory cytokines in the ileum, and ameliorate the blood biochemical indices	[85]
Composite HDPs	200 mg/kg	Feeding	Chickens	– 1.4	– 5.0	+ 3.8	Increased serum antibody levels of H9N2 AIV, improved the development of bursa and thymus	[86]

### Swine

In swine, HDPs have been studied most extensively with weaned piglets. Post-weaning diarrhea is one of the most serious problems for swine producers worldwide. It is usually caused by proliferation of enterotoxigenic *E. coli* (ETEC) in the intestine and is characterized by reduced growth performance and increased mortality of piglets [87, 88]. Supplementation of feed with HDPs can effectively control post-weaning diarrhea and ameliorate the associated adverse effects on weaned piglets [89]. For example, Xiong et al. evaluated the effects of feeding composite HDPs (lactoferrin, cecropin, defensin, and plectasin) to weaned piglets from five different farms. Piglets were feed with dietary supplement of 3 g/kg composite HDPs (mixture of natural lactoferrin, cecropin, defensin, and plectasin) showed decreased incidence of diarrhea (from 9.42% to 5.22%) and increased survival rates (from 93.34% to 97.42%). Average daily gain (ADG) and feed efficiency (G/F) were also significantly improved [90].

Weaned piglets are very susceptible to pathogens and stressors caused by changes in the intestinal flora due to the immature development of the immune system and compromised intestinal integrity. Consequently, effects of HDPs on immunity, intestinal barrier function, and composition of intestinal microbiota are important factors in attenuation of post-weaning diarrhea [91]. Dietary supplementation with microcin J25, an HDP isolated from a fecal strain of *E. coli*, can reduce systematic inflammation by decreasing concentrations of the pro-inflammatory cytokines IL-1β, IL-6, and TNF-α and increasing concentration of the anti-inflammatory cytokine, IL-10, in serum. Compared with the antibiotic colistin sulfate, microcin J25 also decreased the population of *E. coli* and increased the abundance of *Lactobacillus* and *Bifidobacterium* in the feces of weaned piglets [71]. Wu et al. [72] tested the effects of cecropin on piglets challenged with ETEC. They found that supplementation with cecropin significantly reduced the incidence of diarrhea and improved growth performance, similar to the effects observed with antibiotic supplementation (kitasamycin and colistin sulfate). In addition, both cecropin and antibiotics improved nitrogen retention, dietary energy digestibility, intestinal morphology, and intestinal microbiota of challenged piglets. Serum concentrations of IgA and IgG were higher in animals supplemented with cecropin than those treated with antibiotics. These responses indicate that cecropin may have superior performance compared to antibiotics because it can modulate host serum immune responses [72]. Effects of HDPs on immunoglobulin levels of weaned piglets were also reported by Yuan et al. They found that a mixed HDP (swine defensin and a fly HDP) increased serum concentrations of IgG, IgM, IgA, and the classical swine fever antibody CH50 in weaned piglets in a dose-dependent manner, suggesting that HDPs can improve humoral immunity of weaned piglets [73]. In addition to dietary inclusion, injection of the HDP WK3 (a linear trpzip-like β-hairpin HDP composed of 14 amino acids) alleviated diarrhea in piglets, increased villus height in the ileum, reduced cecal abundance of *Enterobacterium *spp., and attenuated intestinal oxidative damage caused by ETEC [74]. Intraperitoneal injection of cathelicidin-BF (isolated from *Bungarus fasciatus* in China) suppressed intestinal inflammation by inhibiting the NF-κB signaling pathway and enhancing immune cell phagocytosis via STAT-1 [88], improved the intestinal barrier function by increasing expression of tight junction proteins, including zonula occluden-1, occludin, and claudin-1 in the jejunum and colon of weanling piglets [75].

Deoxynivalenol (DON) is a mycotoxin produced by certain *Fusarium* species that often contaminates corn, wheat, oats, barley, rice, and other cereals in the field or during storage [92]. It is a serious threat to animal production worldwide, especially for pigs [93]. Ingestion of high concentrations of DON can cause intestinal injury, reduce feeding efficiency, and suppress growth; in serious cases, it causes emesis, rectal bleeding, and diarrhea [94]. HDPs can protect weaned piglets from the toxic effects of DON [76]. Composite HDP (primarily antibacterial lactoferrin peptides, plant defensins, and active yeast) supplementation has been shown to mitigate growth inhibition and oxidative damage and to repair DON-induced intestinal injury in weanling piglets. This improvement may be a result of the ability of HDPs to enhance immunity, intestinal morphology, epithelial cell proliferation, and intestinal protein synthesis of weanling piglets [76].

In a MRSA-challenged pig model, HDPs showed favorable therapeutic effects. Huang et al. reported that injection of 2.5 mg/kg body weight epinecidin-1 (derived from *Epinephelus coioides*) completely protected pigs against death caused by MRSA after one week, decreased pathogen counts in multiple organs, enhanced serum levels of the proinflammatory cytokines IL-6, IL-1β, and TNF-α [77].

### Poultry

*E. coli*, *Salmonella*, and *Clostridium perfringens* are commonly found in poultry. These pathogens cause intestinal inflammation, diarrhea, epithelial damage, systemic sepsis, and death in severe cases. HDPs reportedly reduce bacterial pathogen load and increase the abundance of beneficial bacteria in the intestine, which alleviates negative effects on broilers [70]. Daneshmand et al. tested the effects of cLF36 (an HDP extracted from camel lactoferrin) on *E. coli*-challenged chickens. Dietary supplementation with cLF36 increased the abundance of *Lactobacillus *spp*.*, decreased harmful bacteria in the ileum, and up-regulated genes associated with immune cells and tight junction proteins [78]. Supplementation with microcin J25 mitigated negative effects in broilers challenged with *E. coli* and *Salmonella*, mainly by decreasing populations of total anaerobic bacteria and *E. coli* in the feces, improving villus height in the duodenum and jejunum, and reducing concentrations of IL-6, IL-1β, and TNF-α [79]. Necrotic enteritis is a well-known enteric disease in broilers that is induced by *Clostridium perfringens*. Supplementation with the HDPs cLFchimera and sublancin ameliorated necrotic enteritis-related intestinal lesions and reduced growth inhibition in broilers challenged with *Clostridium perfringens*. These HDPs improved intestinal morphology and restored the balance of microbiota in the ileum and cecum by decreasing and increasing the abundance of *Clostridium *spp. and *Lactobacillus *spp*.*, respectively [80, 81]. Moreover, cLFchimera also positively affected expression of cytokines, tight junction proteins, and mucin in the jejunum [80].

Heat stress causes a variety of physiological disturbances, such as systemic immune dysregulation, intestinal injury, endocrine disorders, and reduced antioxidant capacity [95]. Chickens are vulnerable to heat stress because their thick feathering and absence of sweat glands minimizes their capacity to reduce body heat [96]. Hu et al. evaluated the effects of swine gut host defense peptides on chickens under chronic heat stress. They found that supplying chickens under heat stress with SGAMPs improved ADG, G/F, villus height, gut mucosal thickness, the number of intestinal intraepithelial lymphocytes and goblet cells, and concentrations of IgA in the small intestine [82]. The protective effects of dietary plectasin on boilers were also observed by Ko et al. in tropical environmental conditions. Supplementation with plectasin increased goblet cell counts in the jejunum and ileum and increased the serum concentrations of IFN-γ and IgY [83]. These studies indicated that HDPs may alleviate the adverse effects of heat stress by improving intestinal health and influencing immunomodulatory responses of the intestinal mucous and the innate and humoral immune systems.

HDPs could also be supplemented as growth promoters or immunomodulators in the absence of bacterial challenge or heat stress. Choi et al. reported that dietary supplementation of HDP-A3 linearly improved ADG, retention of dry matter, dietary digestibility of energy and protein, and intestinal villus height compared with non-supplemented birds [84]. Compared to treatment with enramycin, plectasin improved ADG and G/F and enhanced levels of antibodies against Newcastle disease virus (NDV) and H9N2 avian influenza virus (AIV) in yellow-feathered chickens [85]. Compared with enramycin zinc bacitracin, treatment with combined HDPs (plectasin and cecropins) showed positive effects on growth performance and serum antibody levels of H9N2 AIV and improved development of the bursa and thymus [86].

### Ruminants

The rumen is an essential organ in ruminants, which produces short chain fatty acids and essential amino acids and vitamins by microbial fermentation [97]. Methane is also a significant product of rumen fermentation, accounting for 2%–12% of gross energy lost from feeds [98]. Methane is a potent greenhouse gas, and methane emissions from agriculture represent 40% of total anthropogenic emissions, with the largest single contributor (25%) being enteric fermentation in ruminants [99, 100]. Some host defense peptides, especially bacteriocins, can inhibit *Methanococcus vannielii*, *Methanobacterium*, and *Methanomassiliicoccus luminyensis* through several different mechanisms [101, 102]. In an in vitro experiment, bovicin HC5 (a bacteriocin from *Streptococcus bovis* HC5) reduced methane production by 50%, even at low concentrations. Cultures gradually lost their ability to produce methane after treatment with bovicin HC5. Methane was not detected after four transfers, suggesting that ruminal methanogens could not quickly adapt and evolve resistance to bovicin HC5 [103]. Another in vitro study compared the effects of monensin and nisin on rumen fermentation and microbiota. Nisin showed greater effects in reducing methane production and acetate/propionate ratios than monensin did, indicating that nisin is a potential alternative to monensin for ruminants [104]. Although the roles of HDPs in suppressing methane production are documented in vitro, additional in vivo experiments are needed to determine the specific roles of HDPs in ruminant production.

HDPs have also been reported to show antibacterial activity against pathogens that induce bovine diseases such as bovine mastitis, bovine respiratory disease complex, and bovine viral diarrhea. Peptide H18R (H2) can be internalized into MAC-T cells and inhibit MRSA. H2 showed greater efficiency than vancomycin in controlling S*. aureus,* which causes mastitis. In a mouse model of *S. aureus* E48-induced mastitis, H2 reduced the bacterial load in the mammary glands and alleviated both histopathological damage in mammary tissues and polymorphonuclear neutrophil infiltration of alveoli, demonstrating that H2 can be used as a therapeutic agent to treat *S. aureus*-induced mastitis [105]. The cow mammary gland also secretes HDPs, such as psoriasin, cathelicidin and lactoferrin, which play different roles for local defense against bacterial infection in the mammary gland [106].

*Mycoplasma bovis* is an important contributor to the bovine respiratory disease complex. Bovine NK-lysin-derived peptides can damage the plasma membrane and kill *M. bovis* [107]. Małaczewska et al. compared the effects of nisin, lysozyme, lactoferrin, and combinations of these compounds against bovine viral diarrhea virus (BVDV) in vitro. All of the tested HDPs showed anti-BVDV effects. The combination of nisin and lactoferrin was the most potent in reducing extracellular viral titer and intracellular viral RNA levels [108]. These studies performed both in vitro and in animal models highlighted HDPs as promising new candidates for the treatment of bovine diseases.

Although many HDPs have been used as feed additives for pigs and chickens, only a few studies have evaluated their use in ruminants. Liu et al. and Ren et al. tested the effects of mixed HDPs (swine defensin and a fly antibacterial peptide) on goats. They found that mixed HDPs improved rumen microbial community structure by increasing *Fibrobacter*, *Anaerovibrio*, *Succiniclasticum*, and the ciliate genus *Ophryoscolex*, while simultaneously decreasing *Selenomonas*, *Succinivibrio*, *Treponema*, and the ciliate genera *Polyplastron* and *Entodinium*. Xylanase, pectinase, and lipase showed increased activity and acetic acid, propionic acid, and total volatile fatty acids were present at higher levels in the rumen after dietary treatment with HDPs [109, 110]. These results indicated that HDPs could be used as feed additives for goats to improve growth performance. However, additional feeding experiments should be conducted to evaluate the effects of HDPs supplementation on ruminants.

## Challenges and prospects

In recent years, many HDPs have been used as antibiotic alternatives in animal feeding and shown beneficial effects on farm animals. However, the application of HDPs in animal production still faces some challenges.

### Preparation of HDPs

The preparation of HDPs has limitations including low yield, high cost and conditions for activity maintenance, which limit their large-scale production and application in animal production. Currently, the methods for HDPs preparation mainly include: biological material extraction, chemical synthesis and gene engineering expression [111]. Although HDPs exist widely in organisms, their content in biological tissues is low and so the separation is difficult. Chemical synthesis can get a certain number of samples, however, the error rate and side reaction increase with the increase of the molecular weight of HDPs, and the cost is quite high. Gene engineering expression may be the most economical method to obtain large quantities of HDPs at present. However, exogenous expression of HDPs is more difficult than other peptides because they are easily attacked by proteases, and the more intractable problem for recombinant *E. coli* expression system is the toxicity for bacteria cells as well as bacterial LPS contamination. To overcome these problems, HDPs are often expressed by means of fusion proteins or hybrid peptides.

The common tags for fusion expression include thioredoxin (Trx), glutathione-stransferase (GST), maltose-binding protein (MBP) and small ubiquitin-like modifier (SUMO) et al. [112]. Meng et al. [113] expressed plantaricin as a fusion protein with Trx in *E. coli* BL21 (DE3) with a yield up to 9–11 mg/L, and purified plantaricin showed strong antimicrobial activity against *Micrococcus luteus*, *Staphylococcus epidermidis*, *Lactococcus lactis*, *Lactobacillus paracasei* and *Listeria innocua*. Cao et al. [114] successfully expressed broad spectrum of antibacterial peptide proSP-B (rat lung surfactant protein B precursor) by fusion with GST in *E. coli* pLySs, which showed low toxicity to *E. coli*. Lamer et al. [115] designed a His6-SUMO-peptide-intein system to express lactococcin A, leucocin A, faerocin MK, neopetrosiamide A in *E. coli* BL21(DE3), which protected these DHPs against degradation, and also improved yields (up to 17-fold) compared with standard expression and isolation procedures. However, fusion proteins are usually needed to be removed to release activated peptides, which increases the difficulty and cost of HDPs preparation.

Hybrid peptides refer to HDPs fused to other HDPs or functional proteins to provide bifunctional properties [116]. Sun et al. [117] combined bovine lactoferrin (LfcinB) and human lysozyme (hLY) in *Pichia pastoris* GS115 expression system, and the results showed that the antibacterial activity of hybrid peptides LfcinB-hLY against *E. coli* K88 was higher than that of hLY and LfcinB solely, and was not effected by trypsin and chymotrypsin digestion. Liu et al. [118] reported that hybrid peptide cecropinA-thanatin had broad-spectrum antimicrobial activity without hemolysis and good stability in vitro as well. However, the properties and application potential of hybrid HDPs need to been deeply investigated each.

In sum, developing appropriate expression system and perfecting the expression strategy would remain a challenge in this field.

### Stability of HDPs

Many HDPs are susceptible to the digestion of endogenous proteases, such as trypsin and pepsase in digestive tract, which result in low efficiency at the site of action and limit their administration by oral or water. In addition, temperature, pH and salt concentration can also change the structure of HDPs and affect the interaction of HDPs with pathogens. Strategies such as amino acid substitution, peptide cyclization, peptide chain modification as well as encapsulation with nanoparticles have been used to improve metabolic stability, conformational stability and bioavailability of HDPs [119, 120].

Amino acid substitution is popular to improve peptide stability against protease digestion, including *D*- or unnatural amino acids residue substitution [121, 122]. For example, Lu et al. [123] synthesized derivatives of the cationic HDP Pep05 (the putative active domain of histatin 5) by substituting *L*-amino acid residues with *D*- and unnatural amino acids, such as *D*-lysine, 4-aminobutanoic acid, and the results showed that both improve the stabilities of the peptides toward proteases. Nonetheless, it should be noted that the cost of synthetic peptides containing *D*- and unnatural amino acids is higher than solely of *L*-amino acids. Cyclization enabled peptides to have a more rigid conformation and partly shield the potential protease-scissile sites at the free termini and backbone of peptides, therefore improved the protease stability. The type of peptide cyclization mainly includes four categories: head-to-tail, head-to-sidechain, sidechain-to-tail and sidechain-to-sidechain. But the outcome of peptide cyclization may depend on sequence diversity and the complicated structure of HDPs, which cannot be easily predicted [124, 125]. The common peptide chain modifications are amidation, acetylation, methylation, PEGylation, lipidation and glycosylation [120]. For example, C-terminal amidation of HDP-N6 (a variant of arenicin-3) enhanced its ability to penetrate the bacterial and stability toward trypsin, as well as reduced hemolysis [126]. C-terminal PEGylation of pig-derived HDPs protegrin-1 exhibited more efficient antibacterial activity and higher stability toward trypsin degradation [127]. Encapsulation with nanoparticles for HDPs delivery may provide another strategy to improve drug bioavailability and safety, avoid enzymatic degradation, enhance controlled release and prevent aggregation [128]. Lai et al. [129] reported a self-assembling peptide nanoparticles remained largely intact after 8 h of degradation by proteases, demonstrating the proteolytic stability of the self-assembling peptide nanoparticles. Nonetheless, the stability of HDPs encapsulated with nanoparticles should be eventually evaluated in vivo.

### Safety of HDPs

The absorption and metabolism of most HDPs in vivo are rarely reported. Whether HDPs could be degraded or absorbed by intestinal tract? How are HDPs metabolized by the body? What are the effects of their metabolites on the body? These questions are still not been explained clearly. Therefore, potential toxicity of HDPs, such as immunogenicity and hemolysis in vivo should not be ignored. Additional efforts are required to explore the pharmacokinetics and pharmacodynamics of HDPs.

## Conclusions

The broad-spectrum antibacterial activities of HDPs have been widely demonstrated, making them promising alternatives to antibiotics. The immunomodulatory properties of HDPs mean they likely have superior performance compared to antibiotics in production of livestock. It is reported that HDPs could improve growth performance, intestinal health, and immunity of farm animals. However, problems on preparation, stability and safety of HDPs still limit their large-scale application. With the in-depth study of HDPs and the development of biotechnology, these challenges to HDPs will be figured out for better application of HDPs in animal production.

## Data Availability

Not applicable.

## Abbreviations

HDPs: Host defense peptides
AMPs: Antimicrobial peptides
MRSA: Methicillin-resistant *Staphylococcus aureus*
VREF: Vancomycin-resistant *Enterococcus faecal*is
*E. coli*: *Escherichia coli*
LPS: Lipopolysaccharides
TA: Teichoic acids
GPCRs: G protein-coupled receptors
hDP: Human beta-defensin
FPRL1: Formyl peptide receptor-like 1
IL: Interleukin
TNF-α: Tumor necrosis factor-α
TLR4: Toll like receptor-4
MAPK: Mitogen-activated protein kinase
NF-κB: Nuclear factor-κB
MD-2: Myeloid differentiation factor-2
CCR2: C–C chemokine receptor type 2
MCP: Monocyte chemoattractant protein
CXCL1: Chemokine ligand 1 protein
MDC: Macrophage-derived chemokine
VEGF: Vascular endothelial growth factor
GM-CSF: Granulocyte–macrophage colony-stimulating factor
MIP: Macrophage inflammatory protein
ETEC: Enterotoxigenic *E. coli*
ADG: Average daily gain
G/F: Gain:feed
DON: Deoxynivalenol
NDV: Newcastle disease virus
BVDV: Bovine viral diarrhoea virus
AIV: Aavian influenza virus
Trx: Thioredoxin
GST: Glutathione-stransferase
MBP: Maltose-binding protein
SUMO: Small ubiquitin-like modifier
LfcinB: Bovine lactoferrin
hLY: Human lysozyme
